# Supplementary material for: Big dairy data to unravel effects of environmental, physiological and morphological factors on milk production of mountain-pastured Braunvieh cows
Source: R Soc Open Sci. 2020 Jul 1;7(7):200638. doi: 10.1098/rsos.200638 (PMC7428251; doi:10.1098/rsos.200638)
Supplement: Sup. Mat. S1 [file rsos200638supp1.pdf]

Crit	Calving month	Δmilk alp	Δmilk total	Δd	p-value
Lact #	9	5.3	16.9	0.0070	1.16E-01
	10	7.0	17.5	-0.0036	5.74E-01
	11	11.3	18.8	-0.0071	4.55E-04
	12	16.2	19.8	-0.0077	1.69E-03
	1	18.9	19.4	-0.0066	4.61E-01
	2	21.4	19.8	-0.0080	1.00E+00
Pregnancy stage	9	23.2	2.5	0.0333	2.16E-15
	10	18.4	4.3	0.0278	1.98E-20
	11	12.1	4.1	0.0250	1.40E-27
	12	6.4	3.6	0.0203	6.84E-18
	1	2.5	2.9	0.0161	1.63E-04
	2	1.0	2.8	0.0202	1.93E-01
THI-3d	9	3.2	0.2	0.0065	1.78E-01
	10	1.8	0.3	0.0039	1.17E-01
	11	1.3	0.4	0.0030	1.28E-01
	12	0.1	0.1	0.0004	1.00E+00
	1	0.8	0.6	0.0023	5.38E-01
	2	1.5	1.7	0.0046	4.96E-02
THI-30d	9	0.1	0.0	0.0002	1.00E+00
	10	2.3	0.3	0.0049	3.57E-02
	11	1.9	0.5	0.0044	5.24E-03
	12	0.8	0.4	0.0022	3.10E-01
	1	0.6	0.5	0.0018	1.00E+00
	2	1.0	1.1	0.0030	5.30E-01
CSI-3d	9	-1.3	-0.1	-0.0026	1.00E+00
	10	-0.1	0.0	-0.0002	1.00E+00
	11	-0.7	-0.2	-0.0017	1.00E+00
	12	-0.4	-0.2	-0.0011	1.00E+00
	1	-0.5	-0.4	-0.0015	1.00E+00
	2	-0.8	-0.9	-0.0023	9.23E-01
CSI-30d	9	-0.7	0.0	-0.0014	1.00E+00
	10	-0.2	0.0	-0.0004	1.00E+00
	11	0.2	0.1	0.0005	1.00E+00
	12	0.2	0.1	0.0004	1.00E+00
	1	-0.6	-0.5	-0.0018	9.44E-01
	2	-1.4	-1.6	-0.0043	7.45E-02
Precipitations in sping	9	9.3	0.5	0.0189	3.77E-16
	10	8.4	1.2	0.0181	1.22E-32
	11	7.2	2.0	0.0171	6.47E-44
	12	6.0	2.9	0.0159	3.93E-39
	1	5.5	4.2	0.0159	2.09E-25
	2	5.1	5.8	0.0157	1.52E-19

Crit	Calving month	Δmilk alp	Δmilk total	Δd	p-value
Biogeographical region	9	10.4	0.6	0.0209	3.82E-20
	10	9.2	1.3	0.0198	9.38E-47
	11	8.2	2.3	0.0196	1.69E-73
	12	6.9	3.3	0.0183	3.84E-59
	1	5.3	4.0	0.0152	4.47E-19
	2	5.1	5.9	0.0159	2.37E-11
Altitude	9	5.3	0.3	0.0107	6.72E-07
	10	5.3	0.7	0.0115	1.18E-18
	11	5.6	1.6	0.0133	1.35E-44
	12	4.6	2.2	0.0121	1.10E-37
	1	3.6	2.8	0.0104	2.57E-14
	2	3.7	4.2	0.0115	5.20E-12
Difference in altitude	9	1.2	0.1	0.0025	1.00E+00
	10	1.1	0.2	0.0023	3.30E-01
	11	2.2	0.6	0.0052	2.27E-07
	12	2.6	1.3	0.0070	2.14E-13
	1	3.5	2.7	0.0101	3.84E-15
	2	3.8	4.3	0.0118	2.59E-13
Aspect (100m)	9	0.4	0.0	0.0008	1.00E+00
	10	0.8	0.1	0.0018	6.76E-01
	11	0.3	0.1	0.0006	1.00E+00
	12	0.3	0.1	0.0007	1.00E+00
	1	0.3	0.2	0.0008	1.00E+00
	2	-0.2	-0.3	-0.0008	1.00E+00
Aspect (1km)	9	1.6	0.1	0.0032	5.04E-01
	10	1.1	0.2	0.0025	1.78E-01
	11	0.8	0.2	0.0020	7.99E-02
	12	0.7	0.4	0.0020	8.61E-02
	1	0.5	0.4	0.0015	1.00E+00
	2	0.4	0.4	0.0012	1.00E+00
Height	9	7.7	14.9	-0.0137	7.31E-04
	10	8.5	14.5	-0.0125	4.68E-06
	11	9.9	14.7	-0.0091	3.89E-04
	12	11.5	14.7	-0.0089	9.68E-03
	1	11.2	12.4	-0.0022	1.00E+00
	2	11.9	10.9	0.0105	9.12E-01
Foot angle	9	2.8	6.6	-0.0095	1.32E-02
	10	3.3	6.2	-0.0079	2.60E-03
	11	4.8	7.2	-0.0082	3.21E-03
	12	7.4	8.4	-0.0065	4.39E-01
	1	9.6	9.3	-0.0025	1.00E+00
	2	11.1	9.4	-0.0005	1.00E+00

Sup. Mat. S1: For each criterion and calving month, the between-group difference in milk production in the alp and over the total lactation cycle is reported, together with the Δd (reflecting how differently the two groups are impacted by alping) and its associated significance.
