# Supplementary material for: Big dairy data to unravel effects of environmental, physiological and morphological factors on milk production of mountain-pastured Braunvieh cows
Source: R Soc Open Sci. 2020 Jul 1;7(7):200638. doi: 10.1098/rsos.200638 (PMC7428251; doi:10.1098/rsos.200638)
Supplement: Sup. Mat. S2 [file rsos200638supp2.pdf]

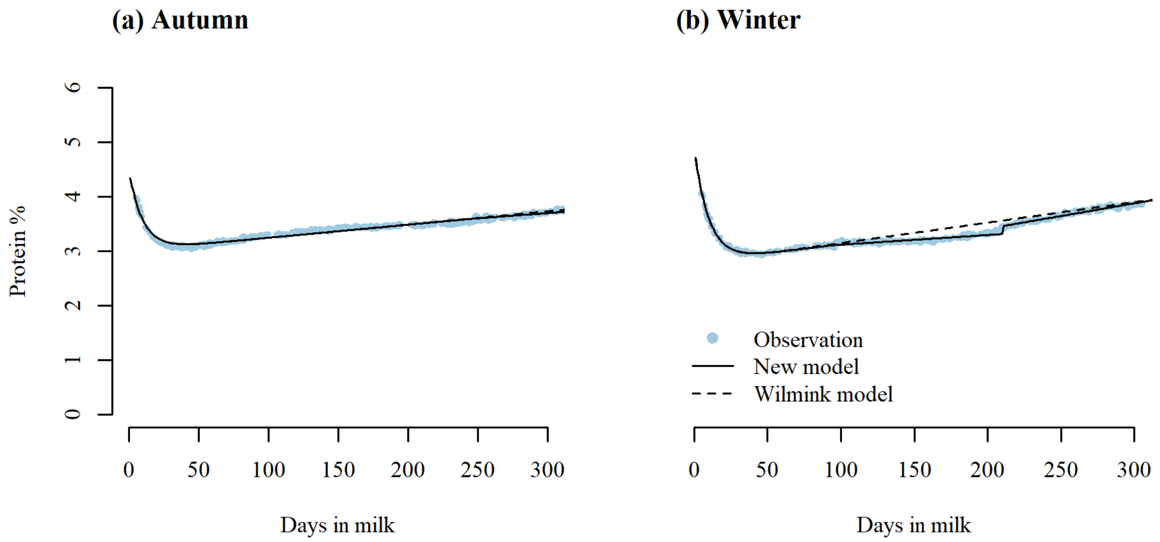

Sup. Mat. S2: Evolution of protein percentage over a lactation cycle as derived from the proposed model (full line) and the Wilmink model (dashed line) for cows that calved in September (a) and February (b). The Wilmink model was fitted using points from the beginning of the curve only, i.e. before alping. Each dot represents the average of milk records per day. When t>245 (a) and between 95 and 210 (b), records from the alp only are used to calculate the average, whilst records from the lowland farm only are included for the remaining time frame. The impact of alping on protein content is considerably smaller compared to its influence on milk yield (Fig. 2)
