# Supplementary material for: Big dairy data to unravel effects of environmental, physiological and morphological factors on milk production of mountain-pastured Braunvieh cows
Source: R Soc Open Sci. 2020 Jul 1;7(7):200638. doi: 10.1098/rsos.200638 (PMC7428251; doi:10.1098/rsos.200638)
Supplement: Sup. Mat. S3 [file rsos200638supp3.pdf]

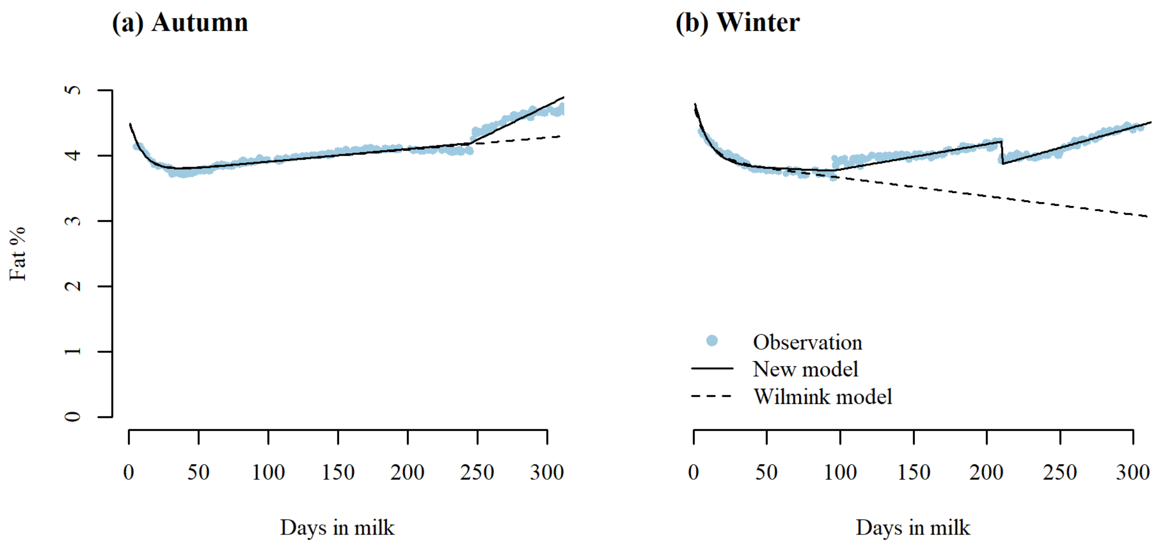

Sup. Mat. S3: Evolution of fat percentage over a lactation cycle as derived from the proposed model (full line) and the Wilmink model (dashed line) for cows that calved in September (a) and February (b). The Wilmink model was fitted using points from the beginning of the curve only, i.e. before alping. Each dot represents the average of milk records per day. When t>245 (a) and between 95 and 210 (b), records from the alp only are used to calculate the average, whilst records from the lowland farm only are included for the remaining time frame. It should be noted that the shape of the Wilmink curve in plot b seems inaccurate, since we typically expect fat content to increase again after the minimum is reached. This is due to the fact that alping starts shortly after the minimum and too few observations are present to model correctly the end of the lactation curve.
