# Supplementary figures and images for: Big dairy data to unravel effects of environmental, physiological and morphological factors on milk production of mountain-pastured Braunvieh cows
Source: R Soc Open Sci. 2020 Jul 1;7(7):200638. doi: 10.1098/rsos.200638 (PMC7428251; doi:10.1098/rsos.200638)

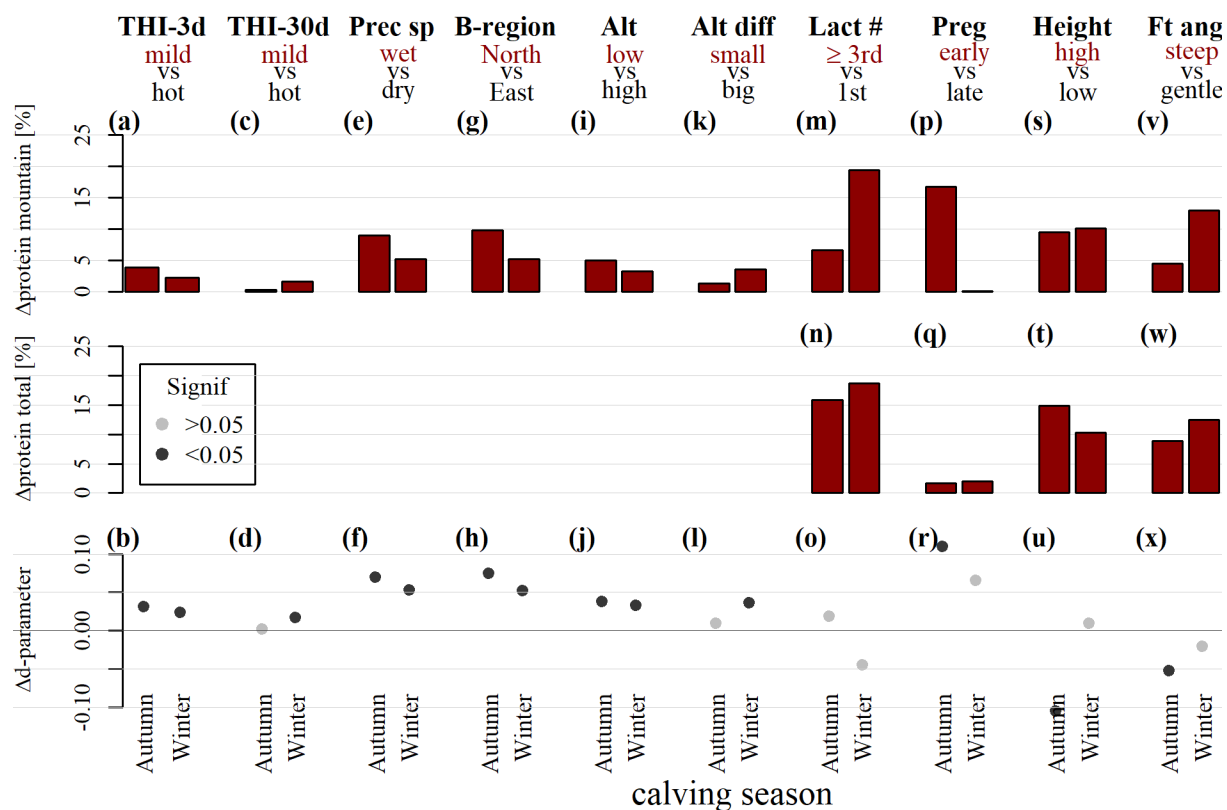

Supplement: Sup. Mat. S4 [file rsos200638supp4.pdf]
