# Supplementary material for: Big dairy data to unravel effects of environmental, physiological and morphological factors on milk production of mountain-pastured Braunvieh cows
Source: R Soc Open Sci. 2020 Jul 1;7(7):200638. doi: 10.1098/rsos.200638 (PMC7428251; doi:10.1098/rsos.200638)
Supplement: Sup. Mat. S5 [file rsos200638supp5.pdf]

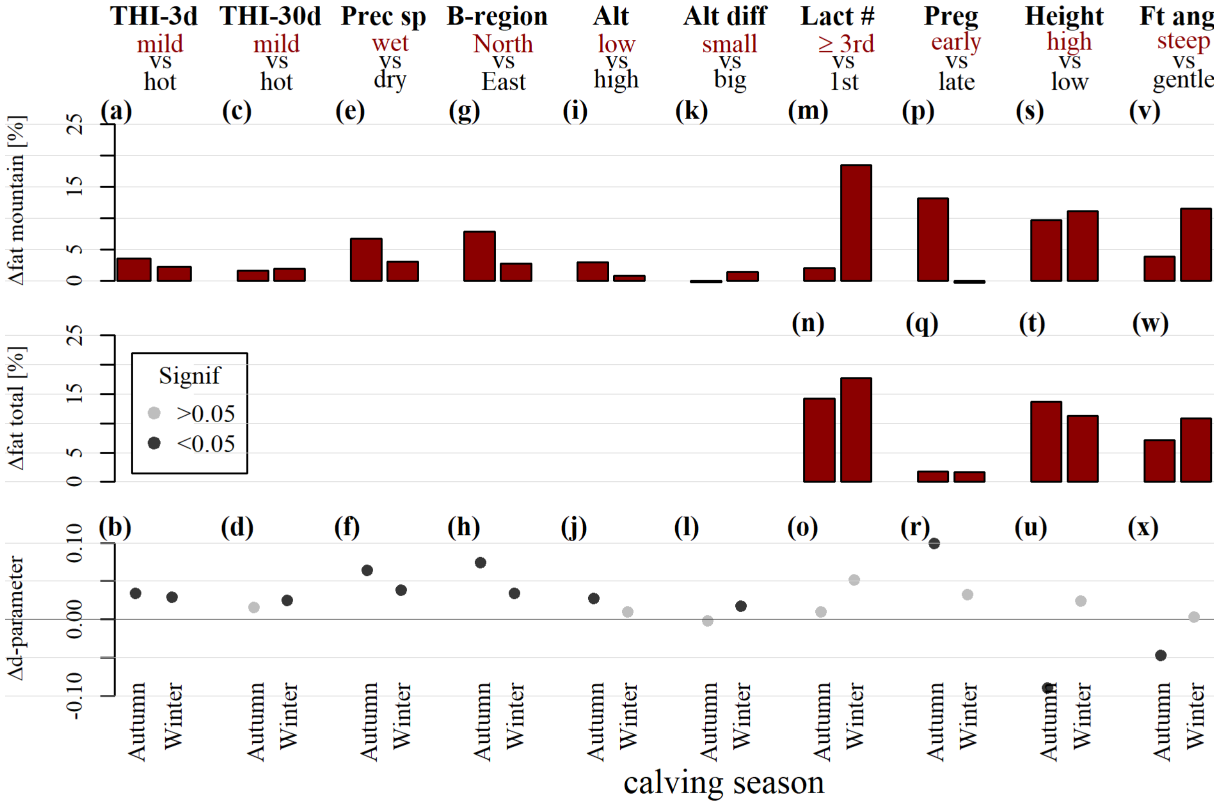

Sup. Mat. S5: The effect of influencing factors is tested by investigating the difference in fat yield (fat content * milk production) between two groups of animals coming from contrasted conditions (first and third tertiles, except for THI where second and third tertile are chosen). Each factor is here reported in a separate column. At the top of each column, the factor name as well as the contrasted groups are reported; the group with highest fat yield during alping is chosen as the reference group, highlighted in red. In each barplot, the first bar shows the result for autumn calving, and the second for winter calving. The between-group difference in fat yield during alping is displayed in the top panel, the between-group difference in fat yield during the whole lactation in the intermediate panel, the change in the d-parameter at the bottom. The Δd-parameter indicates how the reference group is impacted by alping compared to the other group, with positive values meaning lower negative impact (see Eq. 4). Significant Δd values are plotted in black, while grey indicates non-significance. Environmental factors affects production during alping only, making a comparison of the whole fat production redundant (which is why no graph is present in the intermediate panel of the concerned variables).
